# RNA-Seq Reveals Differential Gene Expression in *Staphylococcus aureus* with Single-Nucleotide Resolution

**DOI:** 10.1371/journal.pone.0076572

**Published:** 2013-10-07

**Authors:** Joseph Osmundson, Scott Dewell, Seth A. Darst

**Affiliations:** 1 Laboratory of Molecular Biophysics, the Rockefeller University, New York, New York, United States of America; 2 Genomics Resource Center, the Rockefeller University, New York, New York, United States of America; Institute of Enzymology of the Hungarian Academy of Science, Hungary

## Abstract

*Staphylococcus aureus* is a gram-positive cocci and an important human commensal bacteria and pathogen. *S. aureus* infections are increasingly difficult to treat because of the emergence of highly resistant MRSA (methicillin-resistant *S. aureus*) strains. Here we present a method to study differential gene expression in *S. aureus* using high-throughput RNA-sequencing (RNA-seq). We used RNA-seq to examine gene expression in *S. aureus* RN4220 cells containing an exogenously expressed transcription factor and between two *S. aureus* strains (RN4220 and NCTC8325-4). We investigated the sequence and gene expression differences between RN4220 and NCTC8325-4 and used the RNA-seq data to identify *S. aureus* promoters suitable for *in vitro* analysis. We used RNA-seq to describe, on a genome wide scale, genes positively and negatively regulated by the phage encoded transcription factor gp67. RNA-seq offers the ability to study differential gene expression with single-nucleotide resolution, and is a considerable improvement over the predominant genome-wide transcriptome technologies used in *S. aureus*.

## Introduction

*Staphylococcus aureus* (*S. aureus*) is a pathogenic bacterium that can cause a variety of infections, most notably of the skin [1]. *S. aureus* infections can be difficult and costly to treat due to antibiotic resistance, especially in the Methicillin-Resistant *Staphylococcus aureus* (MRSA) strains [2-4].

High-throughput studies have been particularly useful to examine global gene expression in *S. aureus* [5-7]. The ability to examine the effects of transcriptional modulators on all genes and at multiple time points provides rich data that can be critical in evaluating regulatory networks [7-11]. RNA-seq has become standardized for eukaryotic samples [12,13], but only a relatively small number of prokaryotic species have been examined by this technique. In *S. aureus*, RNA-seq was recently used to identify small non-coding RNAs [14] and to study the role of anti-sense transcription [15], but transcriptome studies in *S. aureus* have largely used microarray techniques to examine global gene expression changes [16,17]. It has been demonstrated in eukaryotic samples that RNA-seq provides data that better matches qPCR [12,13].

*S. aureus* colonizes the nasal cavity of 30% of the human population, but under certain circumstances can invade tissues and cause disease [1]. Given the ability of *S. aureus* to act as both a commensal bacterial and a pathogen, studies have attempted to identify the key pathways regulating pathogenicity in this organism. A regulatory RNA, termed RNAIII, is thought to be the main effector of the switch to pathogenic growth as it controls the expression of secreted toxins. Levels of RNAIII are regulated by the Agr proteins [18,19].

Due to the lack of traditional therapies to treat highly resistant *S. aureus* strains, lytic bacteriophages have been suggested as potential therapeutic agents [20,21] or as the source of novel antibiotic proteins or peptides. Recent work sequenced *S. aureus* phages and identified proteins with antimicrobial activity [22-24]. One such protein, phage G1 gp67, was originally identified as a global RNA polymerase (RNAP) inhibitor [24-26]. Subsequent work showed that this protein (1) binds to *S. aureus* RNAP, but not *E. coli* RNAP, through an interaction with the global housekeeping transcription factor σ^A^; (2) does not block the functions of σ^A^, including DNA recognition and core RNAP binding; but (3) interferes with the interaction between the core RNAP α subunit C-terminal domain (α-CTD) and UP-element sequences that are only required for transcription at a small subset of promoters [27]. Therefore, gp67 specifically inhibits transcription from promoters that require a strong α-CTD/UP-element interaction, including the rRNA promoters. Because robust rRNA transcription is required for logarithmic growth in prokaryotic cells, gp67 blocks normal cell growth, explaining its antimicrobial effect [27].

In this work, we establish an RNA-seq approach to study differential gene expression in *S. aureus* in the competent lab strain RN4220, and between *S. aureus* strains. To identify *S. aureus* genes repressed by gp67, we expressed gp67 in *S. aureus* cells. In addition to the relative gene expression data that would be provided by microarray, we used the RNA-seq data to identify Single Nucleotide Polymorphisms (SNPs) and to quantitatively evaluate the relative levels of gene expression between loci within the same sample. We examined the differences in the transcriptome of *S. aureus* strains NCTC8325-4 and RN4220 and used the RNA-seq data to identify a putative orphan CRISPR element in these strains.

## Materials and Methods

### Strains and plasmids

RN4220 was obtained from Peter Moyle in Tom Muir’s lab at The Rockefeller University. pRMC2 and NCTC8325-4 were a generous gift from Sivaramesh Wigneshweraraj at Imperial College, London.

### gp67 expression in RN4220

Gp67 was cloned into the *S. aureus* expression vector pRMC2 [28] using primers containing a consensus Shine- Dalgarno sequence and BglII restriction site upstream of the start codon, and a stop codon and EcoRI site downstream. pRMC2-gp67 and empty pRMC2 were then transformed into *S. aureus* strain RN4220 by standard electroporation [29] and transformants were selected on trypticase soy (TS) plates containing chloramphenicol (10µg/ml). RN4220 containing empty pRMC2 and pRMC2-gp67 were grown in TS broth containing chloramphenicol and transgene expression was induced with 100ng/ml anhydrotetracycline, which was the minimum required concentration for maximal cell growth inhibition by gp67.

### RNA purification

RNA was purified from cells at mid-log phase growth (O.D._600_ = 0.4) using the RNeasy kit from Qiagen. Briefly, 2x10^8^ cells were removed from growing cultures, immediately added to 2 volumes of BioStabilize solution (Qiagen) and incubated for 5 minutes at room temperature. Cells were then collected by centrifugation, resuspended in TE buffer containing 1mg/ml lysostaphin and 200µg proteinase K and incubated for 15 minutes at room temperature. 100µl zirconia beads (0.1mm) were added to lyse the cells in a bead beater at top speed for 3 x 2minutes, with a 1-minute rest on ice. The lysate was centrifuged briefly to remove the beads and the remaining procedure was carried out to the manufacturer’s specifications. Purified RNA was quantified using a spectrometer (NanoDrop).

### RNA-seq: Sample preparation and sequencing

**Figure 1:**
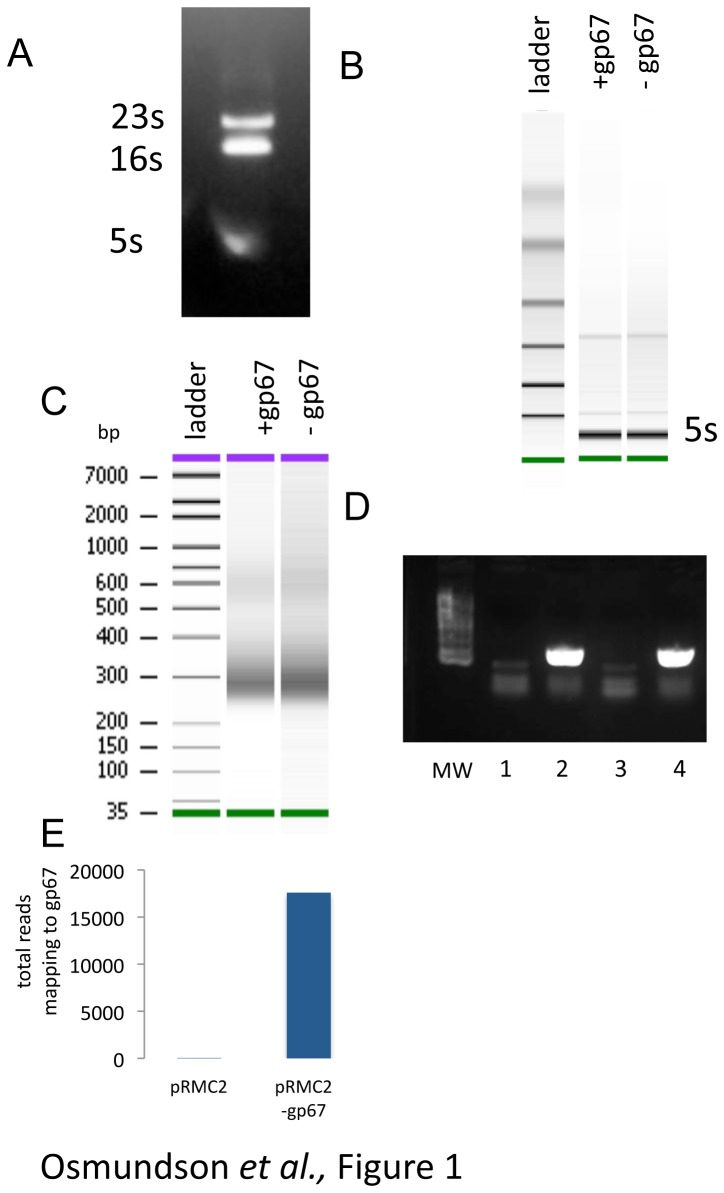
Schematic of RNA-seq in *S. aureus*. a) Total RNA is purified from cells and verified for integrity on a 1% agarose gel. b) rRNA reduction is used to remove the large (16s and 23s) rRNA species from the sample. RNA was assessed by running the samples on a BioAnalyzer. c) After rRNA reduction, the standard Illumina random-prime technique was used to prepare a cDNA library for sequencing. DNA was assessed by running the samples on a BioAnalyzer. d) To verify the representation of mRNA in the cDNA library, and that the prepared samples differed predictably, we performed a PCR for cDNA corresponding to gp67. A band corresponding to gp67 cDNA is only present in cells containing pRMC2-gp67 (lane 4) and not control cells containing pRMC2 alone (lane 3). e) RNA-seq reads mapping to the gene for gp67. RNA-seq reads mapping to gp67 are only present in the RNA-seq data from cells containing pRMC2-gp67 and not control cells containing pRMC2 alone.

RNA was processed as described in Figure 1. Briefly, RNA quality was assessed by visualization on an agarose gel. RiboZero rRNA removal kit for gram-positive organisms (Epicenter) was used to eliminate the 16s and 23s rRNAs prior to sequencing analysis. RNA quality was then evaluated on a BioAnalyzer (Agilent) chip prior to cDNA library synthesis. cDNA libraries were prepared by standard techniques for subsequent Illumina sequencing using the mRNA-seq Sample Prep kit (Illumina) eliminating the step for mRNA amplification. After the rRNA reduction, RNA was fragmented and used as a template for a randomly primed PCR. After the amplification, ends were repaired and ligated to Illumina adapters. The cDNA library was then verified for appropriate fragment size (200-300bp) on a BioAnalyzer chip.

Samples were amplified onto flowcells using an Illumina cBot and sequenced on an Illumina HiSeq2000 for 51 cycles per manufacturer protocols. Raw sequencing data was processed using the onboard SCS/RTA software, yielding 51bp reads.

### RNA-seq: Data analysis

Sequencing reads were processed using TopHat [30], an alignment package designed to align sequencing reads derived from transcribed RNA. The program aligns reads to a reference genome, identifying regions of coverage that correspond to transcribed RNA. These regions are joined and queried for potential junctions by attempting alignment of reads that did not initially align. Reads aligning to multiple locations are kept (to a maximum of 20 potential positions) to assist constructing gene models for genes with repetitive or low complexity features. When aligning reads, 2 mismatches to the reference (Ensembl S_aureus_nctc_8325.EB1.fa) were allowed.

Alignments reported from TopHat were processed by the Cufflinks software package [31] to determine differential expression of genes and transcripts between conditions.

Alignments were quantitated against the Ensembl annotation: (S_aureus_nctc_8325.EB1_s_aureus_nctc_8325.gtf).

Expression values are reported as fragments-per-kilobase-of-gene-per-million-mapped reads (FPKM). Data were visualized using the Integrated Genomics Viewer [32].

Transcripts were quantified by assessing the total number of reads for the entire transcript using the program cuffdiff, part of the Cufflinks suite of tools for sequencing-based transcript assembly and quantification. Briefly, reads were assigned to transcripts as described above and the samples to be compared were evaluated for variance and tested for differential expression. P-values (Tables S1-S6) were determined, and significance was assessed by conducting Benjamini-Hochberg correction for multiple testing [31].

RNA-seq data have been submitted to GEO (accession number GSE48896).

### In vitro transcription assays

In vitro transcription assays were performed as described [25].

## Results

### Development of RNA-seq for gene expression studies in *S. aureus*

To determine the effects of the G1 phage-encoded transcription factor gp67 on all *S. aureus* promoters *in vivo*, we used RNA-seq to examine differential gene expression in *S. aureus*. We cloned gp67 into an inducible expression cassette (pRMC2) [28] and transformed pRMC2 and pRMC2-gp67 into electrocompetent *S. aureus* RN4220 cells to create RN4220-pRMC2 and RN4220-pRMC2-gp67. Addition of inducer to media inhibited growth of cells containing pRMC2-gp67, as previously described, but had no effect on cells containing only empty vector [24,27]. RNA was purified from RN4220-pRMC2 and RN4220-pRMC2-gp67 cells as described in the Materials and Methods.

RNA-seq techniques are standardized in eukaryotic samples [31]. Because the majority of RNAs purified from cells are large, structured ribosomal RNAs (rRNA) (Figure 1a), the mRNA signal must be enriched. In eukaryotic samples, polyA tailed mRNAs are amplified using a polyT oligo [33], but this approach is not applicable to prokaryotic samples. We used a kit developed for the removal of the large bacterial rRNAs (16s and 23s rRNAs) from gram-positive organisms. After rRNA reduction we visualized our samples on a BioAnalyzer (Figure 1b). The small structured RNAs (5s rRNA and tRNAs) remain after the rRNA reduction and comprise the prominent band in the RNA profile (Figure 1b). To prepare a cDNA library for sequencing, we used the standard Illumina random-prime PCR technique (Figure 1c) typically used for mRNA enriched eukaryotic samples.

To ensure that our cDNA library contained mRNA in addition to the small structured RNAs that remained after rRNA reduction, we tested for the presence of gp67 specific mRNA from cells containing pRMC2-gp67 and cells containing empty vector (Figure 1d). Only cells expressing gp67 should contain cDNA specific to its this gene. Performing PCR from the cDNA library showed gp67 mRNA in cells containing pRMC2-gp67 (Figure 1d, compare lane 3 and lane 4), arguing that our cDNA library represents mRNA purified from *S. aureus* cells and that our two samples differ predictably.

The cDNA library was then sequenced using Illumina technology. Before analyzing the data, we searched for RNA reads that mapped to the gene for gp67. We only identified RNA reads mapping to gp67 from the sample containing pRMC2-gp67 (Figure 1e). We then mapped all RNA reads to the *S. aureus* NCTC8325 annotated genome sequence. While the sequence for RN4220 is available, it differs from NCTC8325 only by 121 SNPs and several indels that cluster around phage insertion sites. The NCTC8325 genome is more fully annotated and more amenable to use by the software required to map RNA reads and compare expression levels.

### RNA-seq reveals differential gene expression due to a phage transcription factor

To understand the effects of gp67 on global transcription levels, we searched for genes that were differentially expressed in pRMC2-gp67 cells. gp67 is known to bind to *S. aureus* RNAP and inhibit cell growth [22,24,27]. Fewer than 4% of all transcripts were significantly repressed (p<0.05) by gp67 expression, while another 5% were significantly stimulated (p<0.05). Overall, the vast majority (^≈^91%) of transcripts were unchanged (p>0.05) in cells expressing gp67 compared with control cells containing only empty vector. This targeted effect of gp67 is in agreement with structural and biochemical data [27]. The full list of genes found to be significantly repressed or stimulated upon gp67 induction are listed in Tables S1 and S2 respectively.

### Identification of *S. aureus* promoters using RNA-seq data

RNA-seq analysis, like microarrays, reports only the steady state level of RNA in cells. Additional sample preparation is required to identify primary transcripts [34] or to map RNAP location in the genome under different conditions [35]. Our analysis cannot differentiate between transcripts directly affected by gp67 through its interaction with *S. aureus* RNAP and those indirectly affected by disruption of other regulatory factors or alterations in mRNA stability and degradation. We therefore sought to directly test gp67 at *S. aureus* promoters shown to be susceptible to inhibition *in vivo*. However, very few *S. aureus* promoters have been examined *in vitro* [18,19,36] and the promoters previously examined *in vitro* were not modulated by gp67 expression *in vivo* [27]. Identifying promoter sequences in a genome can be a challenging computational problem. We therefore searched for promoters using the additional information provided by the RNA-seq analysis.

**Figure 2:**
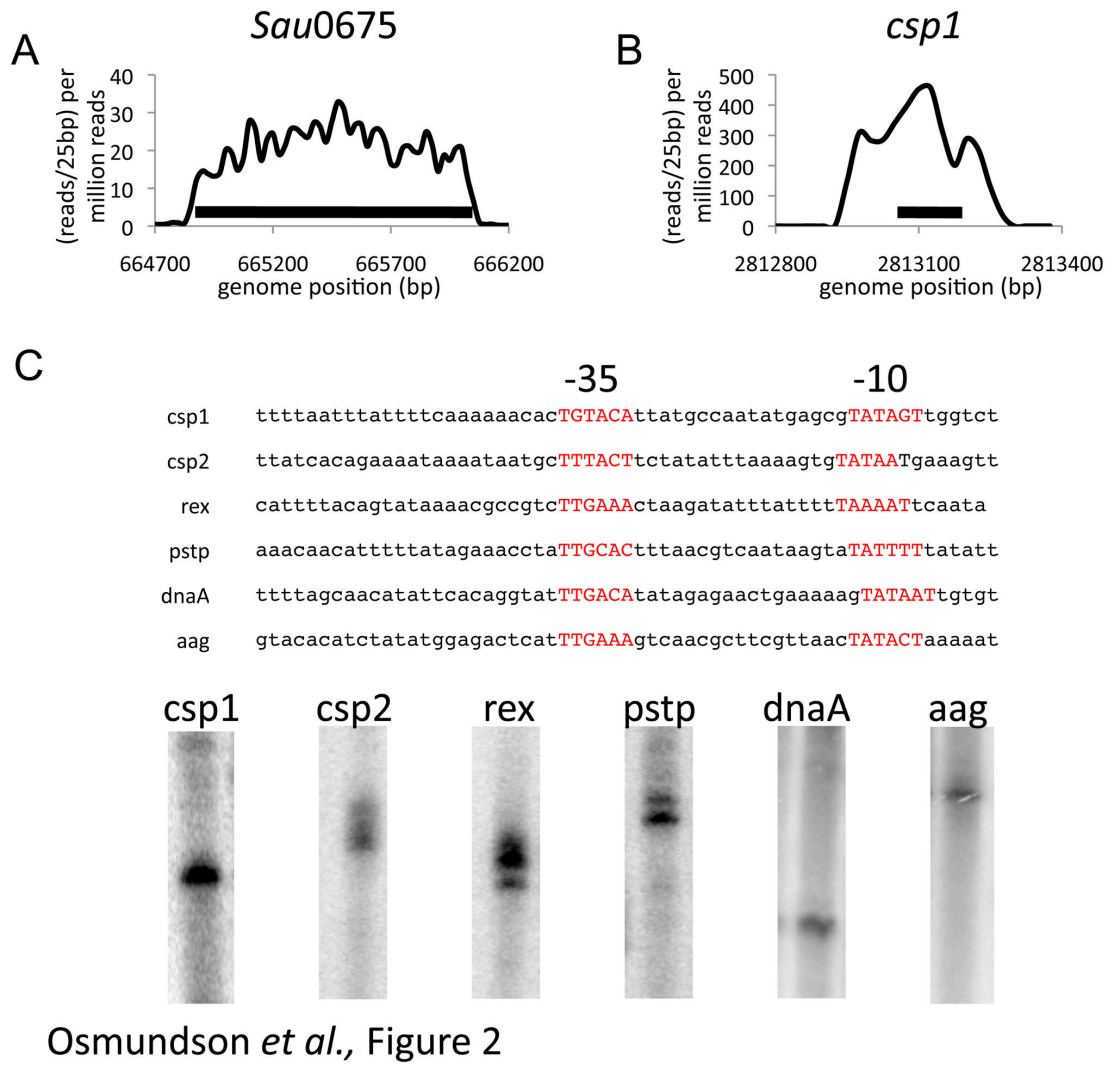
RNA-seq data used to identify promoter sequences in *S. aureus*. a) The NCTC8325 gene *S*. *aureus*0675 shows RNA-seq reads mapping to just upstream of the ATG start codon, but contains promoter-like sequence in this region. Data is represented as reads over 25bp per million total reads and the x-axis shows the position along the *S. aureus* 8325 genome. The black bar represents the coding sequence of the *S*. *aureus*0675. b) The cold shock protein gene (csp1) shows clear evidence for RNA-seq reads upstream of its start codon. Data is represented as in a, with the black bar representing the csp1 coding sequence. c) *S. aureus* promoters identified with the aid of RNA-seq data. Upper panel: Sequence of the promoter elements. -35- and -10-elements are highlighted in red. Lower panel: *in* *vitro* transcription assays show RNA polymerase activity at promoters identified using RNA-seq data.

mRNA processing enzymes can remove 5’ and 3’ UTRs from mRNAs in cells. Mapping promoter start sites requires enriching for primary transcripts that have not undergone processing *in vivo* [34]. For our analysis, we sequenced mRNAs from cells without subsequent enrichment for primary transcripts. Much of our RNA-seq data shows evidence of processing, with RNA-seq reads mapping to just upstream of the start codon for a predicted gene (Figure 2a). Generally, there is no obvious putative promoter element immediately upstream of these transcripts, suggesting that the transcription start site is further upstream and the mRNA has been processed *in vivo*. However, some mRNAs in our data show clear evidence for a long 5’ UTR (Figure 2b). Moreover, many of these transcripts have strong putative promoters (-35 consensus: TTGACA; -10 consensus: TATAAT; ideal spacing: 17bp) just upstream of the mapped 5’ end of the mRNA. This information is not provided by standard microarray analysis that reports only on RNA expression within the coding region.

Promoters identified through the RNA-seq analysis, as described above, were tested for transcription activity using a *S. aureus in vitro* transcription system [27]. These promoters showed robust activity *in vitro* and gave RNA products of the expected size on a urea-PAGE gel (Figure 2c; lower panel). We used this method to identify likely promoter sequences upstream of genes repressed by gp67 expression *in vivo* for subsequent *in vitro* analysis. We showed that gp67 directly inhibited RNAP activity at promoters upstream of genes repressed by gp67 expression *in vivo* [27]. The RNA-seq data, which allowed us to map putative *S. aureus* promoters likely to be directly inhibited by gp67, was critical to our subsequent analysis of the mechanism of RNAP inhibition by this phage encoded transcription factor [27].

### Comparison of relative gene expression between genes

Because RNA-seq gives a direct measurement of numbers of RNA reads per base per million total reads, without relying on hybridization to oligonucleotides, it is more straight forward to quantitatively compare gene expression levels between different loci in the genome. We used RNA-seq data covering the *S. aureus* genome to evaluate which genes are most highly expressed in log-growing cells. Recent work has examined the genomic differences between the commonly used, electroporatable *S. aureus* strain RN4220 [37] and its parent strain NCTC8325-4 [38]. NCTC8325-4 differs from the fully sequenced NCTC8325 by the curing of 3 phage infections [38]. To evaluate the transcriptional differences between NCTC8325-4 and RN4220, and to ensure that RN4220 carrying an empty expression vector was not misrepresentative of baseline transcription in NCTC8325-4, we sequenced RNA purified from NCT8325-4 cells containing no expression vector.

We evaluated the levels of the gene expression in NCTC8325-4 and RN4220. Among the 100 most highly expressed genes, none differed significantly in expression levels between these two strains (Table S3). Similarly, the genes with no evidence for RNA-seq reads were the same between the two strains.

Among the 100 most highly expressed mRNAs in RN4220 and NCTC8325-4, the majority (62) were ribosomal proteins or proteins otherwise involved in translation (the 30 most abundant mRNAs in RN4220 and NCTC8325-4 are shown in Table S3). This is in good agreement with the observation that log-growing prokaryotic cells expend most of their transcriptional resources on supporting translation. Other highly expressed mRNAs corresponded to genes for gylcolysis and sugar metabolism (12), fatty acid biosynthesis (6), chaperones (3), transcription/transcription regulation (3), and redox regulation (3). Nine of the 100 most abundant mRNAs were for conserved proteins of unknown function. The remaining 2 genes were a GTPase required for cell division and a protein translocase.

An additional 212 genes, mostly of unknown function, had no evidence for RNA-seq reads in either NCTC8325-4 or RN4220. Whether any of these genes are upregulated as cells enter stationary phase, or respond to other signals, is unknown but could be evaluated by sequencing RNA from cells grown under various conditions.

### Analysis of Single Nucleotide Polymorphisms between RN4220 and NCTC8325-4

The genome of RN4220 was recently sequenced [37]. In the genome sequence of RN4220, single nucleotide polymorphisms (SNPs) were identified that differ from NCTC8325 and NCTC8325-4. The authors suggested that RN4220 may be deficient in factors required for normal cellular responses to stress and virulence regulation [37]. Additional work characterized SNPs in NCTC8325-4 relative to NCTC8325 [16]. Through our RNA-seq analysis, we can identify SNPs in both the NCTC8325-4 and RN4220 transcriptome, and map these SNPs to the NCTC8325 genome.

**Figure 3:**
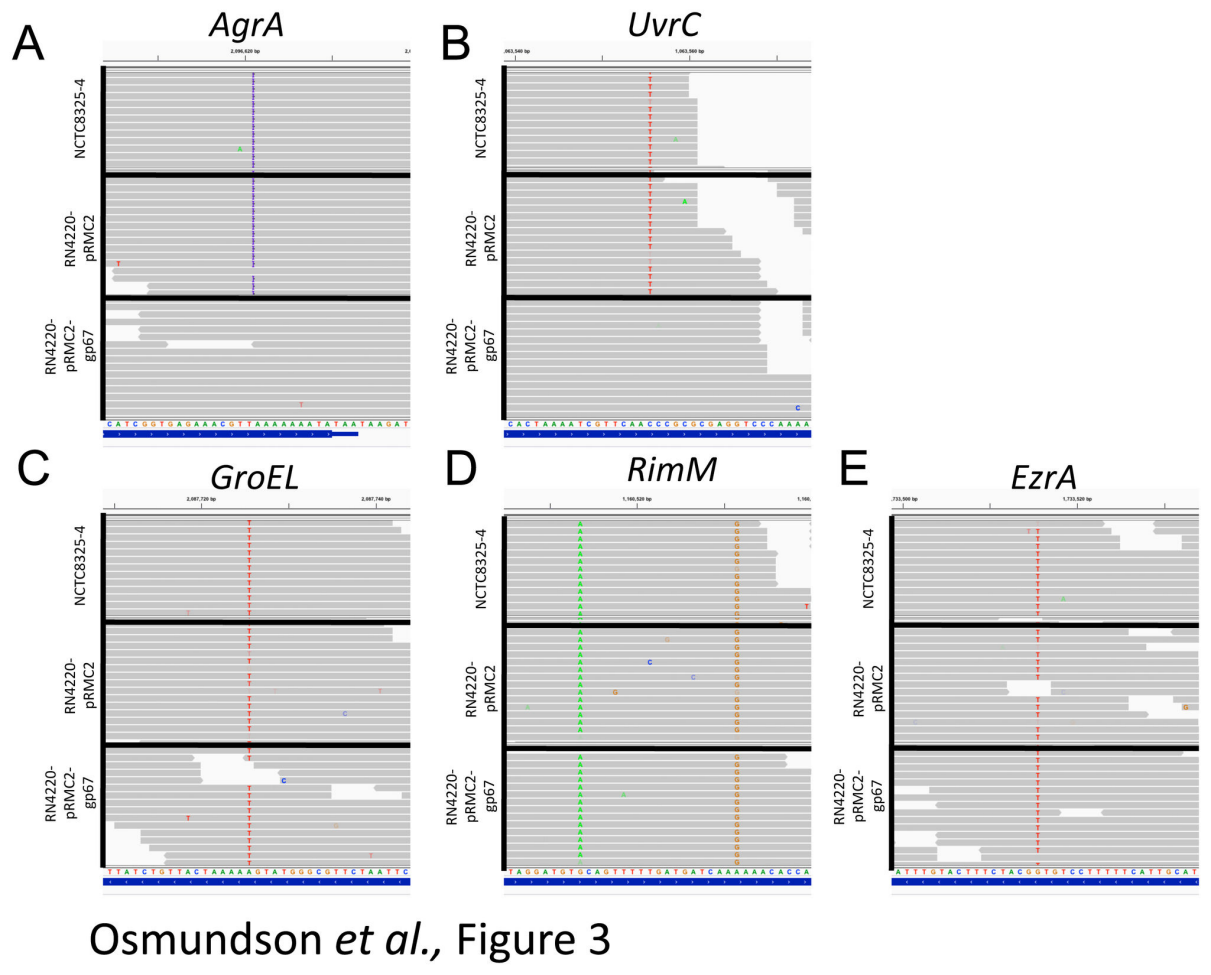
Single nucleotide polymorphisms (SNPs) between RN4220 and NCTC8325-4. a) A previously identified single nucleotide insertion at the C-terminus of the AgrA gene causes a frame shift mutation. The blue bars indicate the single nucleotide insertion, which is present only in RN4220 cells. RNA-seq data was visualized by the Integrated Genomics Viewer (IGV). b) The previously identified non-synonmyous SNP in the UvrC gene is found only in RN4220 cells and not NCTC8325-4 cells. c) A SNP in the GroEL gene that was previously identified in RN4220, but not NCTC8325-4, is identified in both strains. d) Two SNPs previously shown in RN4220 (RimM) are also present in NCTC8325-4. e) A SNP in the EzrA gene that was previously identified in RN4220, but not NCTC8325-4, is identified in both strains.

NTCT8325-4, as analysed by O’Neill (2009), was found to differ from the NCTC8325 genome at 12 locations, and RN4220 had 121 SNPs, including those previously identified in NTCT8325-4. Importantly, our RNA-seq analysis validates several SNPs known to cause functional differences between these two strains as unique to RN4220, such as the frame shift in the virulence transcriptional regulator AgrA (Figure 3a) and the DNA repair factor UvrC (Figure 3b). We also see clear evidence for the G-to-A mutation that causes an early stop codon in *hsdR*, a restriction endonuclease (Table S4). This mutation has been shown to be responsible for the ability of RN4220, and not NCTC8325-4, to accept foreign DNA [39,40]. We do, however, see clear evidence for SNPs previously identified as unique to RN4220 in NCTC8325-4 (Figure 3 c-e). The SNPs in GroEL (Figure 3c), RimM (Figure 3d), and EzrA (Figure 3e), which the authors of the genome sequence of RN4220 argued may effect the fitness of this strain [37], were found in the ancestral NCTC8325-4 genome as well as in RN4220. Roughly half of the additional SNPs identified as unique to RN4220 were similarly found in NCTC8325-4 in our analysis, but not in the analysis done by O’Neill (Table S4). These results were confirmed by resequencing the NCTC8325 genome [41]. These mutations (RimM, EzrA, MurA, and GroEL) are therefore present in the parental strain NCTC8325 and are not unique to either NCTC8325-4 or RN4220.

### RNA-seq reveals differential gene expression between two *S. aureus* strains

The authors of the RN4220 genome sequence argue that the SNPs that differ between the strains may cause functional differences in cellular responses to stress and to the switch to virulent growth [37]. Subsequent work showed that the RN4220 and the parental strain NCTC8325 have similar fitness levels in laboratory conditions [41]. Because RN4220 is electrocompetent and capable of being transformed by expression plasmids, it is well suited to genetic analysis and laboratory studies [29]. We compared gene expression between NCTC8325-4 and RN4220 cells containing pRMC2. While the genomes of all these strains have been sequenced and examined for genomic variations [37,38,41-43], to our knowledge global transcriptional differences have not been examined.

**Figure 4:**
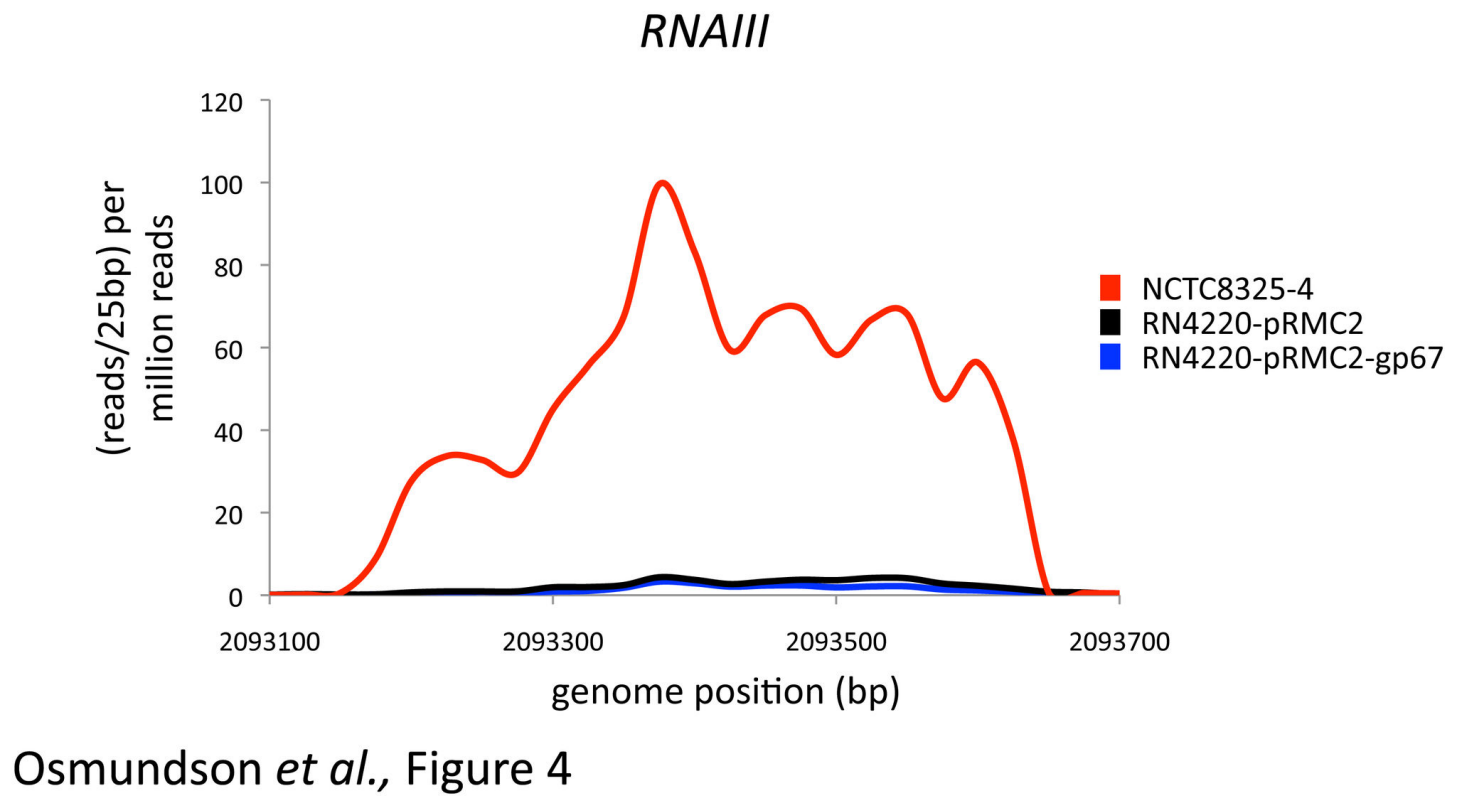
RNAIII is downregulated in RN4220 compared to NCTC8325-4. RNA-seq reads mapping to the gene for RNAIII from NCTC8325-4 (red line), RN4220-pRMC2 cells (black line) and RN4220 cells expressing gp67 (blue line). Data is represented as reads per 25bp per million total reads and the x-axis shows the position in the *S. aureus* 8325 genome. The previously described frameshift mutation in AgrA (see Figure 3a) has been shown to delay the expression of RNAIII in RN4220 cells.

RN4220 has a mutation in the AgrA gene that causes a frame shift near the C-terminus of the protein (Figure 3a). This mutation is known to cause a delayed upregulation of RNAIII, which is a key molecule in the switch to virulent growth [44]. Only four genes are significantly downregulated in RN4220 compared to NCTC8325-4 cells (Table S5a). RNAIII is one of these genes (Figure 4), in agreement with the previous data on the mutation in AgrA [44].

These data show the power of RNA-seq compared to other methods for transcriptome analysis: in one set of data we can identify both the SNP in AgrA that alters its function and the downregulation of RNAIII that is a direct result of this mutation. RNAIII is the most highly repressed gene in RN4220 compared to NCTC8325-4, reinforcing the importance of the mutation in AgrA for regulation at this locus.

The three other downregulated genes in RN4220 (Table S5a) are an acetoactate synthase, which catalyses the formation of 2-acetolactate from pyruvate during stationary phase and an alpha-acetolactate decarboxylase from the same operon. The final downregulated gene encodes a protein of unknown function. Interestingly, four SNPs identified in the RN4220 genome (A-2244467-G, G-2244495-A, and deletions of C-2244932 and T-2244933) all cluster around this gene (2244539-2244724). While these mutations were identified in the RN4220 genome sequence, we see clear evidence for their presence in NCTC8325-4 genome (Table S6). The function of this gene and of these mutations are all unknown.

Thirty-one genes are upregulated in RN4220 carrying an expression cassette and under antibiotic selection compared to NCTC8325-4 cells (Table S5b). Among these upregulated mRNAs, nine encode putative or confirmed ABC transporters. This may be due to the addition of chloramphenicol to the growth media to select for RN4220 cells containing pRMC2; sequencing of RNA from RN4220 cells not containing a plasmid would clarify if this difference is inherent to the strains or rather is a response to the addition of antibiotic to the growth media. ClfB, a clumping factor, is also upregulated in RN4220. This could potentially compensate for the ClfA mutation previously identified in RN4220.

### Identification of a putative orphan CRISPR element in *S. aureus*

Clustered regularly interspaced short palindromic repeats (CRISPRs) are bacterial RNA elements that allow to an adaptive response to phage infection [45]. CRISPRs contain many interspaced repeats that encode a long RNA followed by the Cas genes, which encode the protein machinery required to process the RNA into functional units. After processing, CRISPR RNAs can interact specifically with phage or invasive DNA and induce cleavage [45].

*S. aureus* is not thought to have a functional CRISPR system. No genes in the *S. aureus* genome have any homology to previously identified Cas proteins. Genomic searches for putative CRISPR elements in the *S. aureus* NCTC8325 genome reveal only five weak hits [46].

**Figure 5:**
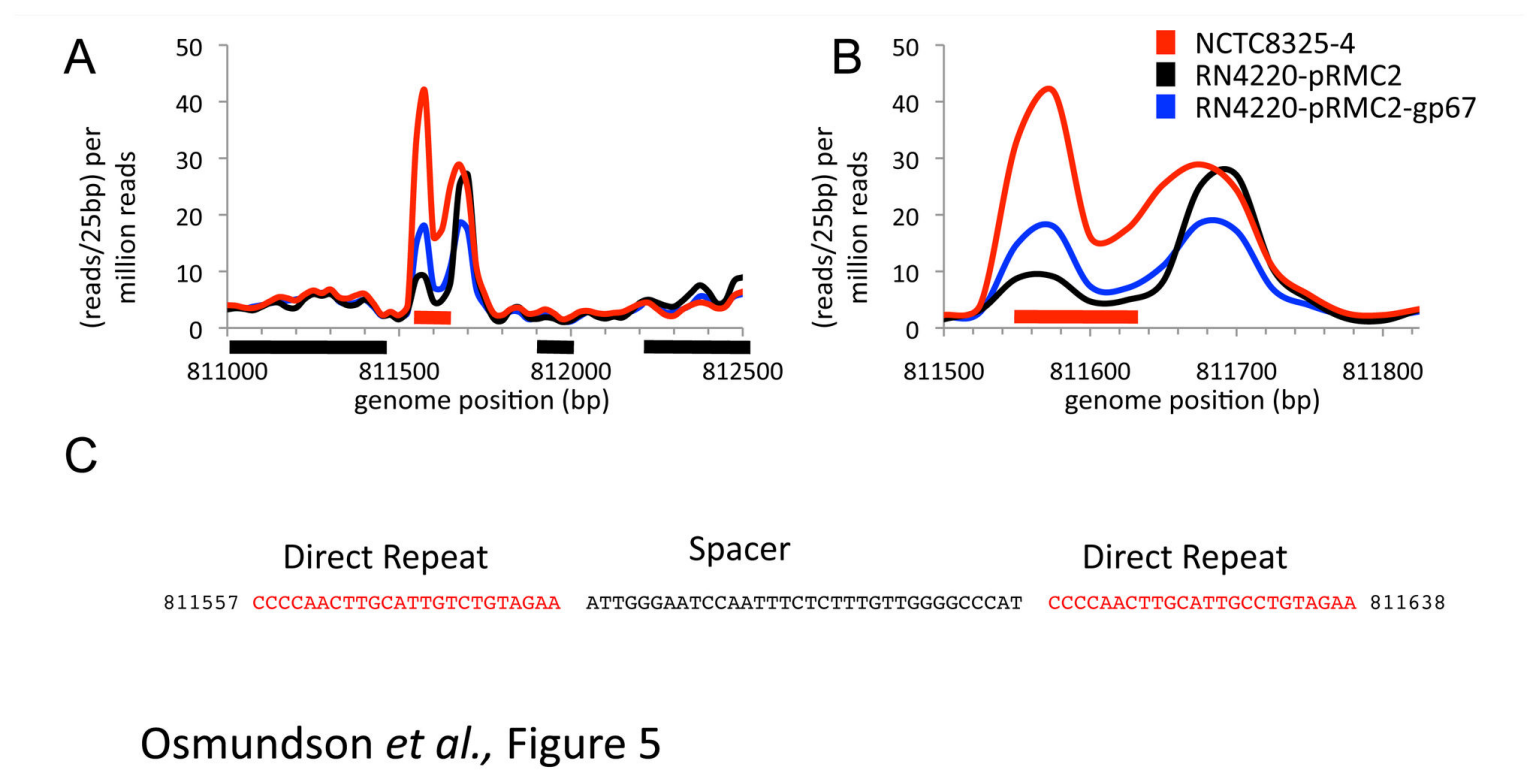
Identification of a putative CRISPR element in *S. aureus*. a) RNA-seq reads mapping to an intergenic region containing a putative CRISPR element. Data is represented as reads per 25bp per million total reads and the x-axis shows the position along the *S. aureus* 8325 genome. NCTC8325-4 (red line), RN4220-pRMC2 (black line) and RN4220-pRMC2-gp67 (blue line) show evidence for RNA-seq reads mapping to the putative CRISPR (red box). ORFs are shows as black box b) Zoomed view of the putative CRISPR element shown as in part a. c) Sequence of the putative CRISPR element with the direct repeats coloured red. The position in the NCTC8325 genome is given before and after the sequence.

We used our RNA-seq data to determine whether RNA was expressed at any of the putative CRISPR loci. While four of the five putative CRISPR elements were located in annotated ORFs, and contained no signal for an RNA element in our RNA-seq data, one putative CRISPR was located in an intergenic region and showed clear evidence for RNA-seq reads (Figure 5). The putative CRISPR had only one repeated unit and had no downstream Cas genes that would be required for active crRNA function [45]. BLAST searches for the CRISPR element revealed that the spacers map to several locations in the *S. aureus* genome including both coding and non-coding regions. This element may be an orphan CRISPR, and reintroduction of Cas genes into *S. aureus* may activate this putative RNA element.

## Discussion

Gene expression and regulation in *S. aureus* is of extensive interest due to the pathogenic importance of this organism [8-11]. A better understanding of the mechanisms through which *S. aureus* switches to its pathogenic transcriptional profile may provide novel targets for drug therapy. Studies in *S. aureus* have used microarray analyses to study differential gene expression in response to exogenously expressed proteins or drugs [7,11,16,17,47]. Here we describe an RNA-seq based approach to study differential gene expression in *S. aureus* both between cells expressing and lacking an exogenously expressed protein and between closely related *S. aureus* strains.

Like microarray analysis, RNA-seq provides relative gene expression levels. We examined the genes downregulated by the expression of a *S. aureus* phage transcription factor, gp67 [24,27]. However, RNA-seq provides additional information on the levels of expression of all transcripts throughout the genome and the sequence differences at single nucleotide resolution. We used the RNA-seq data to evaluate which mRNAs in the *S. aureus* genome are most highly expressed in log-growing cells and to identify novel *S. aureus* promoters for analysis by *in vitro* transcription.

Many studies in *S. aureus* have used the genechip technologies described by Dunman et al. [7]. Dunman et al. used various *S. aureus* strains to validate their genome-wide transcription quantification method. Their work focused in part on the role of ArgA as a transcriptional modulator by using an ArgA knock out strain. RN4220, examined here, contains a mutation in AgrA known to modulate its activity. Like Dunman et al., we show a significant decrease in RNAIII due to ArgA dysfunction. We also find that protein A (*spa*) is upregulated in RN4220, in agreement with microarray analysis of an AgrA knock out strain. However, the transcriptional differences between NCTC8325-4 and RN4220 at mid-log growth appear to be more limited than the differences between wild type RN27 and cells containing an AgrA mutation. We also used RNA-seq data from RN4220 carrying an expression plasmid and NCTC8325-4 to compare gene expression and SNPs between the two strains. Recent work has highlighted potential functional differences between these strains [37,38,41]. Gene expression differences between the two strains were limited, arguing that, during logarithmic cell growth, the two strains are functionally similar [41].

The development of microarray technology in *Staphylococcus aureus*, and the ability to perform genome-wide analysis of transcription under various conditions, increased our understanding of transcriptional networks in this organism. While microarrays are more cost-effective than high-throughput sequencing, the cost of routine RNA-seq experiments have dropped precipitously in recent years, and are likely to continue to drop [48]. RNA-seq provides a more direct output (direct sequencing of RNA molecules) than hybrid-based gene-chip techniques, has been shown to better match qPCR data in eukaryotic samples [12], and allows for a larger dynamic range [48]. Additionally, RNA-seq provides sequence information that is obviously lacking in microarray data, allowing for the identification of SNPs in cells growing under different conditions [33] or in various bacterial strains, as described here. As the costs for high-throughput sequencing continue to drop, RNA-seq may provide unique benefits for transcriptome analyses in various prokaryotic organisms, particularly where gene chips are not available.

The technology presented here could be easily adapted to mechanistic study of transcription in prokaryotes, as has been done in eukaryotic samples [35]. While high-throughput sequencing has been used to map promoter elements in *E. coli* [49], this analysis has not been performed in other organisms to understand the differences between promoter specificity in bacteria [25]. Mapping of 5’ ends and examination of global transcription levels, with single nucleotide precision, under different transcriptional conditions (stationary phase, with expression of various transcription factors or small molecule effectors), which has been recently described in *E. coli* [50], should be expanded to other prokaryotic organisms and growth profiles.

This study shows that RNA-seq is a valuable tool to examine gene expression in *S. aureus*. RNA-seq provides data that was previously only accessible through multiple, complimentary techniques. Because prokaryotic genomes are generally small, and contain relatively short intergenic distances with limited non-coding regions, we sequenced the majority of the *S. aureus* genome through RNA-seq analysis of the transcriptome and were able to identify many SNPs, including in non-coding regions. RNA-seq has become increasingly cost effective and we have developed a protocol for sample preparation in *Staphylococcus aureus* cells. We believe standardization of RNA-seq for prokaryotic samples, and routine transcriptome analysis using high-throughput sequencing, would provide a significant advantage over the current microarray based techniques.

## Supporting Information

Table S1 **Genes significantly downregulated by gp67 expression.** RNA-seq was used to quantify gene expression in the presence and absence of gp67 as described in the Materials and Methods. Cuffdiff was used to quantify gene expression at each loci in the NCTC8325 genome and significance was determined by conducting a Benjamini-Hochberg correction for multiple testing. Only genes at which p<0.05 are listed.
(PDF)

Table S2 **Genes significantly upregulated by gp67 expression.** RNA-seq was used to quantify gene expression in the presence and absence of gp67 as described in the Materials and Methods. Cuffdiff was used to quantify gene expression at each loci in the NCTC8325 genome and significance was determined by conducting a Benjamini-Hochberg correction for multiple testing. Only genes at which p<0.05 are listed.
(PDF)

Table S3 **Most highly expressed genes in NCTC8325-4 and RN4220.** RNA-seq was used to quantify gene expression in the *S. aureus* NCTC8325-4 and RN4220. Cuffdiff was used to quantify gene expression at each loci in the NCTC8325 genome and significance between samples was determined by conducting a Benjamini-Hochberg correction for multiple testing. No genes in this table differ significantly between the two strains.
(PDF)

Table S4 **SNPs identified as unique to RN4220.** SNPs were identified using SAMtools and verified manually be examining the RNA-seq data in Integrated Genomics Viewer.
(PDF)

Table S5 **Genes significantly downregulated (a) and upregulated (b) in RN4220 relative to NCTC8325-4.** **RNA-seq was used to quantify gene expression in the *S. aureus* NCTC8325-4 and RN4220**. Cuffdiff was used to quantify gene expression at each loci in the NCTC8325 genome and significance between samples was determined by conducting a Benjamini-Hochberg correction for multiple testing. Differences of p<0.05 were considered significant and included.
(PDF)

Table S6 **Single nucleotide polymorphisms previously identified as unique to RN4220 but also present in NCTC8325-4.** SNPs were identified using SAMtools and verified manually be examining the RNA-seq data from both NCTC8325-4 and RN4220 in Integrated Genomics Viewer.
(PDF)
