# Supplementary material for: RNA-Seq Reveals Differential Gene Expression in Staphylococcus aureus with Single-Nucleotide Resolution
Source: PLoS One. 2013 Oct 7;8(10):e76572. doi: 10.1371/journal.pone.0076572 (PMC3792026; doi:10.1371/journal.pone.0076572)
Supplement: Table S1 — Genes significantly downregulated by gp67 expression. RNA-seq was used to quantify gene expression in the presence and absence of gp67 as described in the Materials and Methods. Cuffdiff was used to quantify gene expression at each loci in the NCTC8325 genome and significance was determined by conducting a Benjamini-Hochberg correction for multiple testing. Only genes at which p<0.05 are listed. (PDF) [file pone.0076572.s001.pdf]

Table S1

Genome position	Gene function	-gp67[a]	+gp67[b]	fold change[c]	Locus (SAUOHSC)
9754		118.291	21.109	-1.723	0007
12785	trna synthesis	338.936	69.359	-1.587	***serS
78526	Fe transport	320.21	51.904	-1.820	00074 (sirA)
99487		751.303	128.28	-1.768	sodM
164124	Pyrimidine metabolism	109.03	17.41	-1.835	00152 (187)
164748	indolpuyruvate decarboxylate	139.571	21.343	-1.878	00153 (0188)
166582		628.011	93.171	-1.908	ptsG
199582	isoprenyl cystein carboxyl methyltransferase	382.718	86.026	-1.493	00182 (0220)
222778		120.479	20.511	-1.770	0200
271578		431.454	60.837	-1.959	00253 (280)
297407		285.614	47.161	-1.801	00282 (306)
341745		148.352	33.927	-1.475	00328 (0346)
342463		129.186	24.217	-1.674	00329 (347)
342787		168.878	31.855	-1.668	00330 (348)
366132		187.023	41.175	-1.513	00357 (0373)
402507		44.8496	7.0150	-1.855	00399 (set15)
500137	NTP synthesis	742.144	89.702	-2.113	pdxS
501028		841.017	116.51	-1.977	pdxT (sav0520)
536141	2-amino-3-oxobutane from acetyl coa	242.677	39.254	-1.822	00532 (550)
547607		36.7493	7.0773	-1.647	00543.1
551104		507.965	83.640	-1.804	sdrD
690241		195.406	23.006	-2.139	0707
691928	frutcose permease	316.081	52.638	-1.793	00708 (FruA)
722747		1358.73	86.276	-2.757	00738 (SAV0727)
776118		3048	443.72	-1.927	00794 (gapR)
801076	cold shock	5402.94	322.97	-2.817	cspC
804681		207.55	48.308	-1.458	00827 (0824)
806086	028697 acyl transferase	352.807	59.718	-1.776	00830 (0826)
864859	pgi glucose 6-	1297.41	192.49	-1.908	***pgi

	phosphate (required)				
890664		999.459	146.08	-1.923	00919 (0982)
909471		411.913	67.071	-1.815	00937 (1000)
940007		185.156	31.379	-1.775	00963 (1028)
947204		111.982	25.901	-1.464	00973
990820		853.732	147.18	-1.758	01021 (1075)
993034		579.556	64.304	-2.199	01022 1077)
1004297	mrna degredation and processing (required)	966.567	134.26	-1.974	***01036(1089)
1006241		914.677	119.55	-2.035	01037 (def)
1028809		702.261	97.723	-1.972	01064 (pycA)
1033752	heme biosynthesis	527.373	69.373	-2.028	ctaB
1034688		473.681	67.918	-1.942	01067 (1118)
1051882	trna synthetase	281.582	53.940	-1.653	***pheS
1114346	uracil permease	372.335	32.925	-2.426	pyrP
1115681	pyrmadine biosynthesis	409.408	36.638	-2.414	pyrB
1116580	Pyrimidine biosynthesis	413.906	54.705	-2.024	01168 (pyrC)
1117856	Pyrimidine biosynthesis	430.515	85.961	-1.611	01169 (pyrAA)
1122232	Pyrimidine biosynthesis	461.721	87.618	-1.662	01172 (pyrF)
1148092		1135.87	185.42	-1.813	fapR (1228)
1148669	negative regulator of fatty acid biosynthesis	1206.48	210.10	-1.748	plsX
1204422		571.444	71.310	-2.081	01252 (1275)
1242420		1896.92	236.95	-2.080	01285 (glnR)
1242807	glutamine synthetase (required)	2175.49	290.89	-2.012	***01287 (glnA)
1249421		46.319	7.8498	-1.775	1297
1253698		69.4637	10.140	-1.924	01309 SAV1316
1268367	amino acid transporter	597.115	92.257	-1.868	1326
1272662	NTP biosynthesis	304.507	41.202	-2.000	guaC (sav1337)
1273814		458.992	76.713	-1.789	01332 (1338)
1304208	Oxidative stress	874.423	115.66	-2.023	msrA1
1426309	tRNA synthetase	888.695	146.32	-1.804	asnS
1458084		643.869	125.43	-1.636	01505 (1485)
1545327	malonyl co-a fatty acid biosynthesis	525.605	68.133	-2.043	***01624 (accB)

	(required)				
1659667	MreD	238.947	27.886	-2.148	01759 (1648)
1798610	riboflavin biosynthesis	673.018	142.48	-1.553	ribH
1799087	riboflavin biosynthesis	602.566	124.79	-1.575	01887 (ribA)
1800279	riboflavin biosynthesis	647.036	107.00	-1.800	01888 (ribB)
1800918	riboflavin biosynthesis	654.379	115.33	-1.736	01889 (ribD)
1811161	fructose 6 phosphate aldolase	817.344	170.19	-1.569	01901 (1781)
1883879		1006.38	193.93	-1.647	01979 (1847)
1991203	trna synthesitase	953.649	201.14	-1.556	gatC
2103845		593.509	125.44	-1.554	rex 2273 (xcription reg) (2046)
2152964		248.686	53.497	-1.537	02319 2084)
2189428		261.971	54.169	-1.576	02367 (2126)
2256513	Fe transport	477.757	96.856	-1.596	02430 (htsA)
2270735	prostoglandin dehydrogenase, putative	177.138	38.462	-1.527	02447 (2187)
2272019		83.2365	16.023	-1.648	02448 (2188)
2348592		452.331	43.248	-2.347	02554 (2284)
2357498	transcriptional regulators: GntR family	1032.03	200.06	-1.641	sarR
2370059	ribosomal protein E (required)	394.45	42.534	-2.227	***rpsE
2370857		200.504	20.811	-2.265	2580.1
2438501		146.33	33.948	-1.461	02652 (2370)
2453507		181.402	38.899	-1.540	02668 (2385)
2454270		825.607	75.899	-2.387	sarZ
2454932	protein oxidoreductase	533.963	113.27	-1.551	02670 (2387)
2477970		129.194	23.611	-1.700	02694 (2409)
2478588		86.8084	14.415	-1.795	02695 (2410)
2595325		94.3532	15.658	-1.796	02816 (2513)
2619737		92.976	15.579	-1.786	02845 (5326)
2620123	glycolosis	98.7279	11.306	-2.167	02846 ptsG
2691654	Na/SO4 symporter	2120.95	361.13	-1.770	fda (2606)

2801301	Cold shock protein	147.207	27.530	-1.677	03030 (2694)
2813019	trna synthesis	6199.66	634.56	-2.279	cspB

[a] Reads per kilobase per million total reads

[b] Reads per kilobase per million total reads

[c] $= e^{(+gp67/-gp67)}$
