# Supplementary material for: RNA-Seq Reveals Differential Gene Expression in Staphylococcus aureus with Single-Nucleotide Resolution
Source: PLoS One. 2013 Oct 7;8(10):e76572. doi: 10.1371/journal.pone.0076572 (PMC3792026; doi:10.1371/journal.pone.0076572)
Supplement: Table S2 — Genes significantly upregulated by gp67 expression. RNA-seq was used to quantify gene expression in the presence and absence of gp67 as described in the Materials and Methods. Cuffdiff was used to quantify gene expression at each loci in the NCTC8325 genome and significance was determined by conducting a Benjamini-Hochberg correction for multiple testing. Only genes at which p<0.05 are listed. (PDF) [file pone.0076572.s002.pdf]

Table S2

Genome position	Gene function	-gp67[a]	+gp67[b]	(fold change)[c]	locus
42680	trna modification	183.00	1346.6	2.00	39
110292		6.90	31.31	1.51	106
119491	polysaccharide binding prot	4.25	22.63	1.67	114
121648	nadp sugar binding	3.07	14.48	1.55	117
125615	cell wall biosynthesis	4.00	18.92	1.55	120
130861	cell wall biosynthesis	9.13	40.99	1.50	125
133575	cell wall biosynthesis	15.50	66.86	1.46	128
140421		20.74	147.88	1.96	134
141657	ABC transporter	37.82	159.99	1.44	136
190529		33.36	208.49	1.83	174
303758	pfkb carbohydrate kinase	2.41	16.74	1.94	291
308682	sialic acid synthethetase	3.14	17.61	1.72	nanA
330822	MepB (MFS family) transporter	34.68	152.72	1.48	316
337879		4.60	24.69	1.68	325
339970		7.63	38.28	1.61	327
349924	METHIONINE BIOSYNTHESIS	15.25	124.41	2.10	340
365422		26.05	432.25	2.81	356
366695		30.63	660.34	3.07	358
564127	PROLINE TRANSPORT	110.78	589.76	1.67	556
567449	acetyl co-a transferase	24.70	157.40	1.85	558
568931		33.70	187.21	1.71	560
610261		6.33	122.99	2.97	619
614349		9.42	64.79	1.93	624
614928	translation	17.25	86.63	1.61	mnhB2
619570	translation	15.67	95.92	1.81	mnhG2
628913	glycosyl transferase ie WecG / TagA	193.52	1213.98	1.84	640
657303	PHOSPHATE TRANSPORTER	562.03	4493.62	2.08	669
657936	PHOSPHATE TRANSPORTER	533.30	4734.35	2.18	670
688883		226.10	1372.71	1.80	704
716261	sulfate transporter	123.67	1232.61	2.30	731
718985	Histidine biosynthesis	28.07	298.82	2.36	hisC
720394		160.51	912.64	1.74	734
816025		30.42	884.46	3.37	845
855576		298.98	1401.18	1.54	892
889973		196.80	1643.28	2.12	918
901688	peptide transporter	4.00	17.65	1.49	929
908437		5.42	25.51	1.55	936

924132		45.07	435.87	2.27	950
957785	Cystein protease	28.61	132.72	1.53	sspC
960953	Aspartate amino transferase	179.25	1737.92	2.27	989
1025648		289.56	1585.69	1.70	1060
1025817		288.33	1646.66	1.74	1061
1081622	ARG BIOSYNTHESIS	7.08	70.45	2.30	1128
1082646	Carbamate kinase	10.42	73.49	1.95	arcC1
1083816	anion permease	16.52	71.89	1.47	1130
1086146	mallic acid transporter	165.48	891.37	1.68	1133
1087342		126.91	850.30	1.90	1134
1162044		43.21	256.51	1.78	1213
1186353	zinc metallo protease	346.89	2482.23	1.97	1239
1187557	trna synthetase	580.53	4381.59	2.02	proS
1276192		205.49	903.45	1.48	1336
1329326	phosphate transporter	4.43	19.67	1.49	1386
1332693		5.91	68.22	2.45	A01332
1349110		6.26	49.50	2.07	1410
1446046		674.41	5336.69	2.07	gpsA
1447061		593.55	4578.22	2.04	engA
1531152	ArgE, Arg biosynthesis	72.93	438.67	1.79	1606
1596249	ROS scavenging	73.59	361.01	1.59	1686
1635662		35.81	346.15	2.27	1729
1635848		33.56	337.70	2.31	1730
1692301	Co-A biosynthesis	202.54	957.01	1.55	1795
1705673		6.24	40.28	1.87	1804
1706497		10.18	61.67	1.80	1805
1723310		105.23	498.33	1.56	1817
1770560		76.57	813.58	2.36	1864
1795367		8.52	101.50	2.48	1880
1806781		1009.79	8086.02	2.08	1895
1813963		3.74	27.20	1.99	1905
1843315	Serine protease	7.15	32.86	1.53	splE
1844133	Serine protease	4.94	25.96	1.66	splD
1845029	Serine protease	4.11	44.18	2.37	splC
1845806	Serine protease	3.09	29.60	2.26	splB
1846653	Serine protease	2.76	19.67	1.96	splA
1889484		125.92	915.47	1.98	1984
1889998		8.95	45.97	1.64	1985
1978642		18.50	179.01	2.27	2104
1979280		221.48	1459.98	1.89	2106
1980013		202.69	1335.89	1.89	2107
1982200		16.68	295.79	2.88	2109
2031740		71.08	472.18	1.89	hlb
2202995		938.94	14904.3	2.76	2381
2208189	nadp binding prop	9.36	94.24	2.31	2387
2223263		14.04	68.34	1.58	2402
2264436		4.57	24.59	1.68	2437

2266096		44.58	1452.99	3.48	asp23
2266668		14.04	489.08	3.55	2442
2266920		19.98	513.40	3.25	2443
2267631		3.47	72.07	3.03	2444
2279891	Lactose metabolism	71.09	478.15	1.91	lacR
2289533		3.54	33.20	2.24	2466
2290322		33.92	142.67	1.44	2467
2389508		296.07	1437.99	1.58	2600
2391719		41.85	188.11	1.50	2603
2420343		52.11	235.33	1.51	2632
2425298		5.91	68.22	2.45	A02505
2503504	glycerate kinase	85.32	512.81	1.79	2723
2504706		201.33	1558.81	2.05	2724
2516693	NADP binding	176.09	1067.86	1.80	2737
2517700		52.52	797.26	2.72	2738
2519027	drug transporter	392.01	3899.53	2.30	2740
2525681	amino acid transporter	36.93	372.04	2.31	2750
2549036	2-deoxy-D-gluconate 3-dehydrogenase NADP	6.40	29.76	1.54	2772
2590644		28.17	208.43	2.00	2812
2591199		34.47	190.78	1.71	2814
2650766	Squalene synthase	7.71	34.11	1.49	crtN
2652286	Squalene desaturase	5.28	30.00	1.74	crtM
2653186		3.32	20.07	1.80	crtQ
2654319	Phytoene dehydrogenase	2.91	16.62	1.74	crtP
2659919	acetyl transferase	18.93	138.41	1.99	2886
2735411		46.69	299.58	1.86	2973
2775173	Polysaccharide polymerase (biofilm)	7.20	44.80	1.83	icaD
2797472		315.36	1869.31	1.78	3024
2798580	Pyroglutamyl peptidase	30.13	177.12	1.77	pcp
2800599		9.03	54.80	1.80	3028
2816354		210.35	1261.91	1.79	mnmG
2819017	translation	149.09	832.60	1.72	mnmE
2820535	translation	146.23	801.59	1.70	rnpA

[a] Reads per kilobase per million total reads

[b] Reads per kilobase per million total reads

[c] $= e^{(+gp67/-gp67)}$
