# Supplementary material for: RNA-Seq Reveals Differential Gene Expression in Staphylococcus aureus with Single-Nucleotide Resolution
Source: PLoS One. 2013 Oct 7;8(10):e76572. doi: 10.1371/journal.pone.0076572 (PMC3792026; doi:10.1371/journal.pone.0076572)
Supplement: Table S3 — Most highly expressed genes in NCTC8325-4 and RN4220. RNA-seq was used to quantify gene expression in the S. aureus NCTC8325-4 and RN4220. Cuffdiff was used to quantify gene expression at each loci in the NCTC8325 genome and significance between samples was determined by conducting a Benjamini-Hochberg correction for multiple testing. No genes in this table differ significantly between the two strains. (PDF) [file pone.0076572.s003.pdf]

Table S3

Genome position	Gene function	NCTC 8325-4[a]	RN 4220[b]	fold change[c]	p value[d]	locus SAUOHSC
1142973-1143162	translation	21944	19572	-0.17	0.871	rpmB
1345916-1346117	cold shock, putative	18639	18982	0.03	0.979	cspA
520793-521162	translation	14647	15704	0.10	0.925	rplL
1151544-1151778	fatty acid biosynthesis	13995	10009	-0.48	0.624	acpP
518656-519079	translation	13826	19981	0.53	0.622	rplK
529959-530373	translation	13589	15650	0.20	0.849	rpsL
1587819-1587996	translation	13307	8910	-0.58	0.550	rpsU
1201451-1201721	translation	12420	12584	0.02	0.985	rpsO
530438-530909	translation	11783	12623	0.10	0.925	rpsG
1600105-1600357	translation	10753	6378	-0.75	0.439	rpsT
1659192-1659501	translation	10585	10572	0.00	0.999	rplU
1734576-1735179	translation	10422	10869	0.06	0.955	rpsD
2182601-2182856	translation	10254	9372	-0.13	0.895	rpmE2
2627401-2627620	conserved hypothetical	9651	9374	-0.04	0.965	2853
1161540-1161891	translation	9390	8857	-0.08	0.933	rplS
2821009-2821147	translation	9131	6259	-0.54	0.546	rpmH
360629-360926	translation	8764	9270	0.08	0.935	rpsF
2299326-2299725	translation	8524	8664	0.02	0.982	rpsI
360946-361450	translation	8500	8593	0.02	0.988	349
1658570-1658855	translation	8332	8814	0.08	0.935	rpmA
1658866-1659187	conserved hypothetical	8329	9101	0.13	0.897	1756
529607-529862	translation	8262	8630	0.06	0.949	526
1882163-1882508	conserved hypothetical	7817	3588	-1.12	0.250	1977
2299738-2300176	translation	7664	8713	0.19	0.856	rplM
2315349-2316183	translation	7159	8692	0.28	0.794	rplB
1180819-1181588	translation	6897	9291	0.43	0.689	rpsB
1682851-	translation	6236	9140	0.55	0.560	rpmI

1683052						
2361997-2362801	**conserved** hypothetical	6209	3634	-0.77	0.451	2571
1159734-1160010	translation	6076	5102	-0.25	0.790	rpsP
2316215-2317114	translation	6054	6813	0.17	0.859	rpiW

[a] Reads per kilobase per million total reads

[b] Reads per kilobase per million total reads

[c] $= e^{(+gp67/-gp67)}$

[d] Calculated by Cufflinks
