# Supplementary material for: RNA-Seq Reveals Differential Gene Expression in Staphylococcus aureus with Single-Nucleotide Resolution
Source: PLoS One. 2013 Oct 7;8(10):e76572. doi: 10.1371/journal.pone.0076572 (PMC3792026; doi:10.1371/journal.pone.0076572)
Supplement: Table S4 — SNPs identified as unique to RN4220. SNPs were identified using SAMtools and verified manually be examining the RNA-seq data in Integrated Genomics Viewer. (PDF) [file pone.0076572.s004.pdf]

Table S4

Genome position	Putative gene and func	NCTC8325	RN4220	Amino acid change	Locus Tag
33961	SPOUT methyltransferase (rRNA large subunit methyltransferase)	G	A	E/K	SAOUHSC_00027
79054	Periplasmic binding protein	C	T	G/R	SAOUHSC_00074
138265	Aldehyde dehydrogenase	C	T	T/I	SAOUHSC_00132
142082	Conserved hypothetical protein	C	T	P/L	SAOUHSC_00136
174867	HsdR family DNAse	G	A	W/*	SAOUHSC_00162
239805	Non-coding	C	T		
264985	Drug Transporter	C	T	G/D	SAOUHSC_00246
281533	Conserved hypothetical protein	G	A	W/*	SAOUHSC_00262
335921	Conserved hypothetical protein	C	T	S/N	SAOUHSC_00322
388693	Super-antigen like protein	C	T	P/S	SAOUHSC_00383
561510	Glucose 6-phosphate deaminase (putative)	C	T	A/V	SAOUHSC_00552
626575	ABC transporter (putative)	C	T	S/N	SAOUHSC_00637
649126	Conserved hypothetical protein	G	T	None	SAOUHSC_00661
795429	Clumping factor	C	T	S/L	SAOUHSC_00812
920903	Non-coding	C	T		
937981	Conserved hypothetical protein	C	T	None	SAOUHSC_00960
956938	Naphthoate synthase	C	T	None	SAOUHSC_00985
975813	Quinol oxidase AA3	C	T	A/T	SAOUHSC_01002
1063555	UvrC	C	T	P/S	UvrC
1644195	GTP pyrophosphokinase	A	T	I/N	SAOUHSC_01742
1865100	Non-coding	C	T		
1988769	tRNA amidotransferase	C	T	E/K	gatB
2012802	Non-coding	C	T		
2096621	AgrA	*	A	Frame Shift	SAOUHSC_02665
2129629	S1 RNA-binding domain-containing protein	C	T	D/N	SAOUHSC_02297

2212727	Truncated resolve	C	T	D/N	SAOUHSC_02392
2271169	Conserved hypothetical protein	C	T	G/D	SAOUHSC_02447
2318646	Conserved hypothetical protein	C	T	L/F	SAOUHSC_02515
2396232	Imidazolonepropionase	G	A	T/I	SAOUHSC_02606
2408062	Conserved hypothetical protein	G	A	P/L	SAOUHSC_02619
2442934	Non-coding	C	T		
2445548	PTS system sucrose-specific transporter	C	T	A/T	SAOUHSC_02661
2447620	Non-coding	G	A		
2538208	Non-coding	G	A		
2570353	Conserved hypothetical protein	G	A	P/S	SAOUHSC_02798
2603659	Conserved hypothetical protein	G	A	P/S	SAOUHSC_02824
2631998	HMG-CoA synthase	G	A	G/E	SAOUHSC_02860
2685055	2-dehydropantoate 2-reductase	G	A	G/E	SAOUHSC_02920
2701403	Non-coding	G	A		
2813498	Helix-turn-helix domain-containing protein	G	A	P/L	SAOUHSC_03046
