# Supplementary material for: RNA-Seq Reveals Differential Gene Expression in Staphylococcus aureus with Single-Nucleotide Resolution
Source: PLoS One. 2013 Oct 7;8(10):e76572. doi: 10.1371/journal.pone.0076572 (PMC3792026; doi:10.1371/journal.pone.0076572)
Supplement: Table S5 — Genes significantly downregulated (a) and upregulated (b) in RN4220 relative to NCTC8325-4. RNA-seq was used to quantify gene expression in the S. aureus NCTC8325-4 and RN4220. Cuffdiff was used to quantify gene expression at each loci in the NCTC8325 genome and significance between samples was determined by conducting a Benjamini-Hochberg correction for multiple testing. Differences of p<0.05 were considered significant and included. (PDF) [file pone.0076572.s005.pdf]

Table S5a

Genome position	Gene	NCTC 8325-4[a]	RN 4220[b]	fold change[c]	p value[d]	locus (SAUOHSC)
2093157-2093673	RNAIII	1304.97	41.2993	-4.981	0.0000	RNAIII
2244538-2244724	conserved hypothetical	431.246	41.6507	-3.372	0.0002	02416
2290322-2291027	putative alpha-acetolactate decarboxylase	334.645	39.6627	-3.076	0.0006	02467
2291063-2292728	AlsS: Stationary phase metabolism	323.345	41.4389	-2.964	0.0010	02468

Table S5b: Genes significantly upregulated in RN4220 relative to NCTC8325--4 by RNA--seq (listed by fold change)

Genome position	Gene	NCTC 8325-4[a]	RN 4220[b]	fold change[c]	p value[d]	locus SAUOHSC
551104-555154	sdrD	19.6431	593.981	4.9183	0.000	sdrD
141657-142398	putative nitrate transporter	1.51517	44.2299	4.8674	0.000	00136
424709-425735	ABC transporter	3.37815	75.2257	4.4769	0.000	metN1
425738-426398	putative ABC transporter	2.33495	53.3995	4.5153	0.001	00424
896023-898037	putative ABC transporter	3.87502	69.9488	4.1740	0.001	00926
894010-896007	oppB putative	3.58305	62.2538	4.1189	0.001	00924
140803-141316	conserved hypothetical	23.6997	321.892	3.7636	0.001	00135
898055-899711	ABC transporter OppA, putative	8.17295	103.689	3.6652	0.002	00927
1263418-1264699	dhoM (thrC operon)	11.2979	117.789	3.3820	0.005	01320
624571-627080	putative ABC transporter	510.859	6489.18	3.6670	0.008	00636
2722967-2725601	clfB	130.692	1781.62	3.7689	0.009	clfB
1338096-1339086	asd (dap operon)	6.33142	68.3555	3.4324	0.009	01395
547607-550738	hypothetical	22.2434	233.87	3.3942	0.010	sdrC
142411-144144	srpL ABC transporter, putative	3.42187	36.4878	3.4145	0.015	00137
1339087-1340694	amino acid metabolism	9.4465	84.6008	3.1628	0.018	dapA

426434-427277	ABC transporter, putative	12.8884	105.589	3.0343	0.021	00426
1073723-1074074	conserved hypothetical	44.3692	332.065	2.9038	0.023	01115
2484751-2485438	hypothetical	238.492	1921.97	3.0105	0.024	gpmA
73428-74979	cell wall	643.985	7448.74	3.5319	0.027	spa
2794365-2795214	conserved hypothetical, putative SAM adeno transferase	2.12122	20.5846	3.2786	0.027	03021
813119-814833	MetN: Methionine transporter	16.4666	123.011	2.9011	0.030	00843
569401-569569	conserved hypothetical	458.178	3001.92	2.7119	0.031	00561
814850-815672	conserved hypothetical	31.9421	208.052	2.7034	0.032	00844
1736777-1739529	conserved hypothetical	13.7354	96.2486	2.8088	0.032	01832
144156-145188	conserved hypothetical	6.04009	48.4601	3.0041	0.032	00139
276307-280009	conserved hypothetical	15.999	98.0291	2.6152	0.038	00258
2115681-2116686	ilvC, valine / isoleucine metabolism	2.37121	19.9625	3.0736	0.040	ilvC
2581382-2584524	hypothetical	11.1302	63.99	2.5233	0.048	fnbA
2788278-2789097	tRNA synthetase	1.15579	9.79585	3.0832	0.049	hisZ
938929-939460	comK, competence transcription factor	3.63073	29.5025	3.0225	0.049	00961
1264704-1265766	thrC, threonine synthetase	26.608	152.859	2.5222	0.049	01321

[a] Reads per kilobase per million total reads

[b] Reads per kilobase per million total reads

[c] $= e^{(+gp67/-gp67)}$

[d] Calculated by Cufflinks
