# Supplementary material for: RNA-Seq Reveals Differential Gene Expression in Staphylococcus aureus with Single-Nucleotide Resolution
Source: PLoS One. 2013 Oct 7;8(10):e76572. doi: 10.1371/journal.pone.0076572 (PMC3792026; doi:10.1371/journal.pone.0076572)
Supplement: Table S6 — Single nucleotide polymorphisms previously identified as unique to RN4220 but also present in NCTC8325-4. SNPs were identified using SAMtools and verified manually be examining the RNA-seq data from both NCTC8325-4 and RN4220 in Integrated Genomics Viewer. (PDF) [file pone.0076572.s006.pdf]

Table S6

Genome position	Putative genes and functions	8325	8325-4 /RN4220	Amino acid change	Locus tag
22181	---	C	A	---	intergenic
47652	---	T	.	---	intergenic
110019	phosphonate ABC transporter, substrate-binding protein, putative	C	A	16 V-F	00105
392716	---	G	A	---	intergenic
412762	---	.	T	---	intergenic
412765	---	C	G	---	intergenic
590402	Conserved hypothetical protein	G	.	Frameshift	00591
653801	---	C	T	---	intergenic
751285	SecA	A	T	449 E-V	00769
827849	TauE, sulfite exporter	A	T	164 T-S	00860
926213	hypothetical protein	G	C	235 P-A	00952
939304	ComK	C	T	53 E-K	00961
947899	hypothetical protein	C	.	232 P-X	00973
1016979	putative ABC transporter	G	A	220 E-K	01048
1020577	Conserved Hypothetical protein	G	T	286 S-*	01053
1042000	ribosomal protein L32	T	.	34 G-X	01078
1123048	PyrE pyramadine biosynthesis	G	A	42 G-S	01
1160513	rimM	G	A	106 A-T	rimM
1160531	rimM	A	G	112 K-E	rimM
1180886	rpsB 30S ribosomal protein S2	G	.	10 V-X	01232
1283784	hypothetical protein	C	.	943 D-X	01342
1358230	Kdg alpha-ketogluterate decarboxylase	C	T	590 D-N	01418
1562913	rhomboid family protein	A	T	337 *-K	01649
1636255	hypothetical protein	T	.	100 Q-X	01732
1683491	infC translation initiation factor IF-3	T	C	40 K-E	01786
1733515	EzrA	G	T	73 T-N	01827
2087725	GroEL	A	T	218 F-I	Transcript
2106539	ABC transporter, putative	A	T	602 L-F	Transcript
2166163	UDP-N-acetylglucosamine 1-carboxyvinyltransferase	G	C	124 T-R	02337
2221850	hypothetical protein	C	A	244 S-Y	02401
2244467	---	A	G	---	intergenic
2244495	---	G	A	---	intergenic
2244932	hypothetical protein	C	.	296 *-X	02417
2244933	hypothetical protein	T	.	296 *-X	02417

2318272	---	G	A	---	intergenic
2318274	---	G	T	---	intergenic
2318290	---	C	A	---	intergenic 02512 <--> 02515
2349916	---	G	T	---	intergenic
2420618	hypothetical protein;-hypothetical protein	.	T	---	02632;02633
2447620	---	G	A	---	intergenic
2592012	hypothetical protein;-hypothetical protein	A	.	---	02813;02814
2689048	hypothetical protein	G	T	353 V-L	02923
2762204	hypothetical protein	T	C	1514 N-S	02990
2782820	imidazole glycerol phosphate synthase subunit hisF, putative	C	.	247 G-X	03008
649126	hypothetical protein	G	T	202 G-G	00661
841103	hypothetical protein	G	T	39 G-G	00877
841139	hypothetical protein	G	T	51 G-G	00877
1653482	tgt queuine tRNA-ribosyltransferase	G	A	91 I-I	01748
2166183	UDP-N-acetylglucosamine 1-carboxyvinyltransferase	C	A	117 G-G	02337
2383630	hypothetical protein	G	T	68 G-G	02591
2383660	hypothetical protein	G	T	78 G-G	02591
2446246	PTS system sucrose-specific IIBC component	C	T	42 E-E	02662
2446641	hypothetical protein	T	A	95 I-I	02663
2678563	hypothetical protein	T	C	107 P-P	02911
